# Assessing the anti-resistance potential of public health vaporizer formulations and insecticide mixtures with pyrethroids using transgenic *Drosophila* lines

**DOI:** 10.1186/s13071-021-04997-8

**Published:** 2021-09-26

**Authors:** Hang Ngoc Bao Luong, Arunas Damijonaitis, Ralf Nauen, John Vontas, Sebastian Horstmann

**Affiliations:** 1 Institute of Molecular Biology and Biotechnology, Foundation for Research and Technology-Hellas, Heraklion, Greece; 2 Crop Science Division, R&D, Bayer AG, Monheim, Germany

**Keywords:** Fludora fusion, *Drosophila melanogaster*, Deltamethrin, Clothianidin, Transfluthrin, Resistance, Insecticide mixtures

## Abstract

**Background:**

Insecticide resistance—and especially pyrethroid resistance—is a major challenge for vector control in public health. The use of insecticide mixtures utilizing alternative modes of action, as well as new formulations facilitating their uptake, is likely to break resistance and slow the development of resistance.

**Methods:**

We used genetically defined highly resistant lines of *Drosophila melanogaster* with distinct target-site mutations and detoxification enzymes to test the efficacy and anti-resistance potential of novel mixture formulations (i.e. Fludora^®^ Fusion consisting of deltamethrin and clothianidin), as well as emulsifiable concentrate transfluthrin, compared to alternative, currently used pyrethroid insecticide formulations for vector control.

**Results:**

The commercial mixture Fludora^®^ Fusion, consisting of both a pyrethroid (deltamethrin) and a neonicotinoid (clothianidin), performed better than either of the single active ingredients against resistant transgenic flies. Transfluthrin, a highly volatile active ingredient with a different molecular structure and primary exposure route (respiration), was also efficient and less affected by the combination of metabolic and target-site resistance. Both formulations substantially reduced insecticide resistance across different pyrethroid-resistant *Drosophila* transgenic strains.

**Conclusions:**

The use of mixtures containing two unrelated modes of action as well as a formulation based on transfluthrin showed increased efficacy and resistance-breaking potential against genetically defined highly resistant *Drosophila* flies. The experimental model remains to be validated with mosquito populations in the field. The possible introduction of new transfluthrin-based products and mixtures for indoor residual spraying, in line with other combination and mixture vector control products recently evaluated for use in public health, will provide solutions for better insecticide resistance management.

**Graphical abstract:**

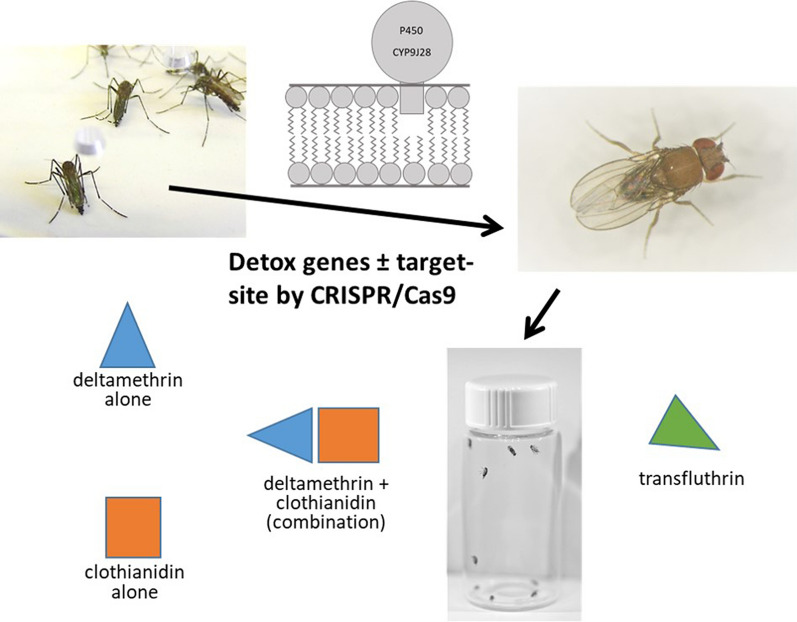

**Supplementary Information:**

The online version contains supplementary material available at 10.1186/s13071-021-04997-8.

## Background

Insect-borne diseases such as malaria and dengue fever cause severe global health and economic problems [1, 2]. Prevention of vector-borne diseases is currently best achieved by vector control which heavily relies on the use of insecticides. For example, malaria cases have been halved since 2000, averting 663 million clinical cases, with > 50% of the reduction due to the use of insecticides [3], primarily pyrethroids. However, insect vectors display a striking ability to develop resistance, with dramatic consequences ranging from shorter residual efficacy time to complete product failure. Many *Anopheles* mosquito populations in malaria-endemic countries are showing striking levels of pyrethroid resistance, and possibly as a result, malaria cases have remained stable or even increased in several places despite the more intense use of insecticides [4–6], while the development of pyrethroid resistance in *Aedes* arbovirus vectors is also on the rise [7].

Changes that alter the amount of toxin that reaches the target site (penetration, activation, metabolism, transport, and excretion) and changes to the pesticide target site (structural changes, knockout, amplification) have been associated with insecticide resistance in insect pests and mosquitoes [8]. Specific mutations in the target site, such as the L1014F (kdr) and the V1016G substitution in the voltage-gated sodium channel (the orthologues to the *para* gene in *Drosophila*), which alter the affinity of pyrethroid binding have been reported in the main vectors of malaria, *Anopheles gambiae*, and dengue fever, *Aedes aegypti*. Cytochrome P450s, such as the *Ae. aegypti* Cyp9J28 [9] and the *Brassicogethes aeneus* P450 Cyp6BQ23 [10], have been functionally implicated in pyrethroid metabolic resistance, respectively.

Combinations of resistance mechanisms drastically increase the resistance impact, often at the operational level [11], and indeed, very strong synergistic interactions of detoxification enzymes and target-site mutations were recently functionally demonstrated in transgenic *Drosophila* [12]. Transgenic *Drosophila* lines expressing distinct pyrethroid-metabolizing P450 enzymes, along with engineered mutations in the voltage-gated sodium channel, displayed substantially greater resistance levels against pyrethroids than the product of each individual mechanism [12].

Strategies for tackling insecticide resistance and for insecticide resistance management (IRM) are officially endorsed by the World Health Organization (WHO) Global Plan for Insecticide Resistance Management in malaria vectors (GPIRM), the nonchemical alternatives within the frame of integrated vector management (IVM), where applicable. In practice, there has been limited experimental evidence for the efficiency of “anti-resistance” potential of new leads and products in public health [13].

A mixture is the concurrent use of two or more insecticides with different modes of action, and its use is based on the hypothesis that the probability of cross-resistance between insecticides is low, while the development of multiple resistance based on different resistance alleles is also low, thus individuals with multiple resistance mechanisms will be very rare. Mixtures are often used in agriculture to control resistant pests or different insect species, but there are very few combination products currently approved for indoor residual spraying (IRS) against vectors of human diseases and limited studies for the evaluation of the efficacy of mixtures [14]. However, combinations of insecticides have been successfully evaluated in bednets; a 2-year large-scale trial in Burkina Faso showed that treating bednets with a combination of chemicals (pyrethroids and the insect growth regulator pyriproxyfen) resulted in reduction in clinical malaria cases, compared with conventional bednets [15]. Another example is the combination of the pyrrole chlorfenapyr with the pyrethroid alpha-cypermethrin, which has been shown to control pyrethroid-resistant mosquitoes [16].

In addition to restoring efficacy, mixtures and combination products may also play a significant role in IRM by delaying the emergence of resistance due to the presence of two or more modes of action in a mix, although more research is needed to validate this approach.

Alternative applications of chemicals belonging to the same chemical class, such as the use of the volatile pyrethroid transfluthrin at ambient temperature in formulations designed to release the compound into an air space, to induce insect behavioural changes (inhibition of host-seeking or killing mosquitoes depending on the chemical concentration in the air space) has also been evaluated [17, 18]. However, knowledge gaps exist, including exact molecular and physiological mode-of-action studies and insights into the relationship between response intensity and insecticide resistance [19].

Here, we tested the efficiency and anti-resistance potential of a novel commercial combination of a pyrethroid and a neonicotinoid insecticide, as well as a formulation of transfluthrin, a highly volatile active ingredient taken up primarily via respiration. Our tests were conducted against genetically defined, highly resistant transgenic *Drosophila* lines with known mosquito target-site mutations and cytochrome P450 detoxification enzymes known to metabolize pyrethroids. Furthermore, our studies explored the value of such transgenic model insects for purposes of screening of vector control assets.

## Methods

### *Drosophila* strains

Four *Drosophila melanogaster* strains representing a spectrum of resistance levels against pyrethroids were chosen from a panel of strains generated and reported in a recent study [12]. These strains harboured a combination of one or more genetic modifications mediating target-site and metabolic enzyme resistance. These modifications included the L1014F or V1016G substitutions in the voltage-gated sodium channel *para* [8] and the heterologous expression in metabolic tissues of the cytochrome P450 pyrethroid metabolizer *Ae. aegypti* Cyp9J28 [9] or *B. aeneus* Cyp9BQ23 [10, 20]. Specifically, strain HR-J28 contained no target-side mutations and constitutively expressed Cyp9J28 under the HR-GAL4 driver [21]. This line was reported to have a low level of resistance to deltamethrin [12] and was used in this study in place of a “wild-type” strain. The addition of the substitution V1016G at the *para* locus in strain HR-J28 resulted in strain V1016G;HR-9J28. Similarly, strain L1014F;HR contained the substitution L1014F at the same locus and the HR-GAL4 cassette but lacked any P450 transgene for heterologous expression. The L1014F-based target-site resistance is already described for several insect species and is found in high frequencies in several *Anopheles* species, for example in *An. gambiae* sensu lato [22]. Crossing the strain L1014F;HR with another strain with the transgene *Cyp9BQ23* (not included in this study) resulted in the strain L1014F;HR-6BQ23, which has both the target-site mutation and constitutive expression of this P450. All flies were reared at 25 °C, 60–70% humidity, and 12:12 h photoperiod on a standard fly medium.

### Insecticides

All compounds were provided by Bayer AG (Leverkusen, Germany) as either liquid or powder formulation (Additional file 1: Table S1). K-Othrine^®^ WG 25 is a powder that contains w/v 25% deltamethrin as active ingredient, while Clothianidin WG 70 has w/v 70% clothianidin. The combination product, Fludora^®^ Fusion WP-SB 56.25, contains w/v 50% clothianidin and w/v 6.25% deltamethrin. These three products are in powder form, and two of them (Fludora^®^ Fusion WP-SB and K-Othrine^®^ WG) are commercially available. The tested transfluthrin formulation is an emulsifiable concentrate (EC) liquid containing 62.5 g active ingredient per litre. All compounds are soluble in water. For contact bioassays, different volatile solvents were used to make appropriate dilutions. At least five different concentrations for each compound were used to determine the LC50 values for the corresponding strain in the corresponding bioassays described below.

### Toxicity bioassays

Tolerance to the compounds was measured by chronic feeding of larvae and acute surface contact for adults, as described previously [12]. For chronic feeding larval bioassay, a cage of 200–300 adults were allowed to lay eggs on agar-juice medium for 24 h. Eggs were collected, washed, and transferred to fresh plates for hatching. First-instar larvae hatched within 24 h were then transferred into vials containing standard *Drosophila* rearing medium mixed with defined concentrations of an insecticide. Control medium for all experiments contained the water used to dissolve the compounds. Five concentrations were used per insecticide, and three to four vials of 50 larvae were collected per concentration per strain. Survival was scored as the total number of pupae present after 12–14 days maintained in a 25 °C incubator in complete darkness, as some compounds were light-sensitive.

For acute surface contact adult bioassays, glass scintillation vials were coated with defined amounts of each insecticide. Control vials were coated with the corresponding volatile solvent. For each vial, 20 non-virgin females (4–5 days old) were collected and allowed to recover from CO_2_ anaesthesia for 24 h before transfer to the appropriate vial. The vials were then plugged with cotton balls that were kept moist with 5% sucrose solution. Survival/mortality was counted after 24 h maintained in a 25 °C incubator without light. Five concentrations were used per insecticide, and five vials of 20 females were used per concentration per strain.

Dose–response analysis was done with ProBit analysis using PoloPlus (LeOra Software, Berkeley, CA) to calculate lethal concentrations of 50% of the population subjected to the experiment (LC_50_ values), 95% limits, and statistical significance of the results.

## Results

### Fludora^®^ fusion is more effective than both clothianidin WG and K-Othrine^®^ WG against highly resistant genotypes in larval bioassays

For all strains tested, Fludora^®^ Fusion WP-SB (deltamethrin + clothianidin) was more effective than K-Othrine^®^ WG (deltamethrin only) (Table 1). Specifically, much lower concentrations/doses of active deltamethrin ingredient in the formulation were required to achieve 50% mortality when Fludora^®^ Fusion was compared to K-Othrine^®^. For example, 0.0033 parts per million (ppm) deltamethrin from the Fludora mixture corresponded to the LC50 of the HR-J28 strain, as compared to 0.79 ppm deltamethrin from K-Othrine^®^ (Table 1). In contrast, amounts of active clothianidin for 50% mortality were similar between Fludora^®^ Fusion (deltamethrin + clothianidin) and Clothianidin WG (clothianidin only) for larval bioassay (Table 1). For example, 0.026 ppm clothianidin from the Fludora mixture corresponded to the LC50 of the HR-J28 strain, as compared to 0.027 ppm clothianidin from K-Othrine^®^ (Table 1).

**Table 1:** Bioassay responses and resistance levels of transgenic *Drosophila* lines against K-Othrine, Fludora^®^ Fusion, and Clothianidin 70 WG in larval feeding bioassays

Genotype	K-Othrine	Equivalent^c^ deltamethrin	RR^b^	Fludora fusion	Equivalent^c^ deltamethrin	Equivalent^c^ clothianidin	RR^b^	Clothianidin 70WG	Equivalent^c^ clothianidin	RR^b^
	LC50^a^	LC50^a^		LC50^a^	LC50^a^	LC50^a^		LC50^a^	LC50^a^	
HR-J28	3.16 (2.76–3.52)	0.79 (0.69–0.88)	1	0.052 (0.02–0.08)	0.0033 (0.001–0.005)	0.026 (0.011–0.043)	1	0.039 (0.034–0.043)	0.027 (0.024–0.03)	1
L1014F;HR	15.008 (12.71–17.51)	3.752 (3.18–4.38)	4.74	0.228 (0.18–0.27)	0.0140 (0.011–0.017)	0.114 (0.09–0.137)	4.38	0.111 (0.093–0.131)	0.078 (0.065–0.092)	2.89
V1016G;HR-9J28	40.58 (32.05–52.99)	10.145 (8.01–13.25)	12.84	0.058 (0.05–0.07)	0.0036 (0.003–0.004)	0.029 (0.025–0.033)	1.12	0.059 (0.054–0.063)	0.041 (0.038–0.044)	1.52
L1014F;HR-6BQ23	48.76 (29.77–91.70)	12.191 (7.45–22.93)	15.43	0.348 (0.30–0.40)	0.0220 (0.019–0.025)	0.174 (0.15–0.198)	6.69	0.191 (0.169–0.213)	0.134 (0.118–0.149)	4.96

^a^Lethal concentration (95% confidence interval) in parts per million (ppm)

^b^Resistance ratio (RR), compared to HR-J28

^c^Equivalent stands for the respective amount of active insecticide ingredient in each pesticide formulation

The resistance ratio (RR) of the highly resistant strains L1014F;HR-6BQ23 and V1016G;HR-9J28 bearing both target-site mutations (L1014F and V1016G, respectively) and pyrethroid-metabolizer P450s (CYP6BQ23 and CYP9J28, respectively) against Fludora^®^ Fusion were 6.69- and 1.12-fold, respectively, compared to the control flies (HR-J28). These values are substantially lower than the respective RR that the same lines showed against K-Othrine (15.43- and 12.84-fold, respectively) (Table 1).

### Transfluthrin EC is more effective than K-Othrine^®^ WG against highly resistant genotypes in adult bioassays

Compared to K-Othrine^®^ WG, the EC formulation of transfluthrin required a significantly lower amount of active compound to achieve the same mortality (Table 2). For example, the resistant strain (L1014F;HR-*6BQ23*) showed LC50 values of 356 µg K-Othrine^®^ WG, but only 0.48 µg EC transfluthrin/vial.

**Table 2:** Bioassay responses and resistance levels of transgenic *Drosophila* lines against deltamethrin (technical), K-Othrine, and transfluthrin EC, in adult contact bioassays

Genotype	Deltamethrin^a^		K-Othrine	Equivalent^d^ deltamethrin		Transfluthrin EC	
	LD50^b^	RR^c^	LC50^b^	LC50^b^	RR^c^	LC50^b^	RR^c^
HR-J28	6.49 (4.051–6.60)	1	10.524 (9.15–12.44)	2.631 (2.29–3.11)	1	0.35 (0.33–0.38)	1
L1014F;HR	39.5 (23.1–53.9)	6.09	331.9 (266.46–40.85)	82.975 (66.61–103.21)	31.54	1.666 (1.336–2.01)	4.76
V1016G;HR-9J28	61.5 (47.5–78.5)	9.48	280.44 (238.46–335.49)	70.711 (59.61–83.87)	26.88	1.98 (1.43–2.25)	5.6
L1014F;HR-6BQ23	233.1 (171.7–333.8)	35.9	1426.224 (1006.24–1934.32)	356.556 (251.56–483.58)	135.5	0.48 (0.11–0.86)	1.36

^a^Data from Samantsidis et al. [12]: topical application of technical deltamethrin, LD50 (95% confidence interval), ng/fly

^b^Lethal concentration (95% confidence interval) in mg/vial

^c^Resistance ratio (RR), compared to HR-J28

^d^Equivalent stands for the respective amount of active insecticide ingredient in each pesticide formulation

The RR of the highly resistant strains L1014F;HR-6BQ23 and V1016G;HR-9J28 against transfluthrin EC was 1.4- and 5.6-fold, respectively, compared to the control flies. These values are substantially lower than the respective RR that the same lines showed against K-Othrine (135.5- and 26.88-fold, respectively) (Table 2).

## Discussion

We used powerful, genetically defined highly resistant *Drosophila* lines with pyrethroid resistance-related target-site mutations and detoxification enzymes which exhibit striking levels of pyrethroid resistance [20, 23], to test the efficacy and anti-resistance potential of the novel active ingredient combination formulation.

Transfluthrin EC was substantially more effective than K-Othrine^®^ WG against highly resistant genotypes in adult bioassays. The demonstration of the efficacy of structurally different pyrethroids, in addition to different primary routes of exposure (volatile transfluthrin vs typical contact pyrethroids such as deltamethrin), against highly resistant flies is in line with previous studies against pyrethroid-resistant *Anopheles funestus* and *Ae. aegypti* mosquito strains [24]. Also, in the transgenic *Drosophila* line expressing CYP6BQ23 (L1014F;HR-GAL4 > UAS-CYP6BQ23), the amount of transfluthrin necessary for 50% mortality is lower compared to deltamethrin (Table 2). The pyrethroid-metabolizing potential of CYP6BQ23 is well known and described for the pollen beetle *Meligethes aeneus* [10], where it mediates the hydroxylation of the phenoxy-benzyl rings of pyrethroids, which is a major reaction step in the detoxification of pyrethroids. As outlined in Horstmann and Sonneck [24], this initial metabolizing step could be inhibited due to the different structure of transfluthrin. It has been shown in *Helicoverpa armigera* that pyrethroids with a tetrafluorobenzyl alcohol moiety are generally less affected by P450 enzymes than others [25]. Additionally, it was demonstrated that multi-halogenated benzyl pyrethroids are more toxic to super-kdr than kdr house flies, another difference between pyrethroid chemotypes observed at the target level [26]. The use of different formulations in glass vials, i.e. on glass surfaces, should minimize an effect resulting from different formulations. A liquid EC formulation, for example, can have disadvantages on porous surfaces, as capillary attraction of small pores on the rough surface affects liquids and pulls them inside. For those surfaces, solid particles (e.g. in SC formulations) or more viscous liquids are suitable. Glass surfaces are largely sealed; therefore, such effects do not particularly occur. However, different formulation components can also influence uptake into the insect. To rule out such effects, further studies should be conducted with identical formulation types or technical active ingredients. Furthermore, the different modes of uptake between pyrethroids are likely to affect efficacy, because transfluthrin exhibits much higher evaporation than deltamethrin. Therefore, the uptake of transfluthrin by the target insects via the insect’s tracheal system will be higher than for the basically non-evaporative deltamethrin, even if the active ingredient vial concentrations are the same. This effect plays a role in the *Drosophila* line L1014F;HR-GAL4, where only the kdr target-site mutation is introduced, but no upregulation of P450 enzymes is induced. The target-site mutation will most likely affect both pyrethroids and will therefore have an affect on transfluthrin as well. However, the resistance ratio of the highly resistant strains bearing both target-site resistance mutations and pyrethroid-metabolizing P450s is substantially lower than the respective resistance ratio that the same lines showed against deltamethrin. Further studies using transgenic *Drosophila* lines can act as role model for the expression of species-related P450 enzymes, for example of *Anopheles* mosquitoes [27], to support the use of transfluthrin in vector control interventions. But as a pyrethroid, the widely distributed target-site resistance at the voltage-gated sodium channel in Africa would still affect the efficacy, especially the L1014F variant. Nevertheless, transfluthrin can be an important additional tool in the control of harmful insects that attack pyrethroids enzymatically at certain positions [24]. The use of such an active ingredient, however, should be in line with other IRM strategies to reduce the pyrethroid-related influence on resistance.

Fludora^®^ Fusion WP-SB performed significantly better than both K-Othrine^®^ WG and Clothianidin WG against highly resistant genotypes. The use of mixtures is efficient and can potentially have a greater impact on IRM. Indeed, a combination of unrelated compounds can (in theory) mitigate the occurrence of resistance and/or delay the selection process of resistant alleles [28]. Insecticide combination products allow the use of at least two active ingredients that the target insect has contact with at the same time. As each of the active ingredients alone can potentially control the insect, they both need to be detoxified for the target insect to survive. After years of using single active ingredient products, this will be an effective alternative in vector control when specific resistance mechanisms for one active ingredient class are unable to degrade the mixture partner at the same time. Therefore, mixture products are also recommended by the WHO GPIRM [29]. In the long run, this will probably select for more general resistance mechanisms, as the selection pressure will still be very high and mixture resistance development has been studied in herbicides already [30]. It underlines the need for additional strategies for resistance management, for example by additional rotational treatment with different mode-of-action products or even with mixture products combining active ingredients that differ from the combination product used as the alternating IRM partner.

## Supplementary Information

**Additional file 1: Table S1.** List of formulations with details about active ingredients, their concentration, physical form, and details of the respective solvent per assay.

## Data Availability

Trial data have been collected and are available at the Institute of Molecular Biology and Biotechnology, Foundation for Research and Technology-Hellas, Heraklion, Greece. Sample materials were produced at Bayer AG, Crop Science Division, Monheim, Germany.

## Abbreviations

EC: Emulsifiable concentrate
kdr: Knockdown resistance
IRM: Insecticide resistance management
WHO: World Health Organization
GPIRM: Global Plan for Insecticide Resistance Management
IVM: Integrated vector management
AG: Stock corporation
LC: Lethal concentration
LD: Lethal dose
WP-SB: Wettable powder in a soluble bag
WG: Water-dispersible granules
CO_2_: Carbon dioxide
RR: Resistance ratio
SC: Suspension concentrate
