# Supplementary material for: Assessing the anti-resistance potential of public health vaporizer formulations and insecticide mixtures with pyrethroids using transgenic Drosophila lines
Source: Parasit Vectors. 2021 Sep 26;14:495. doi: 10.1186/s13071-021-04997-8 (PMC8474913; doi:10.1186/s13071-021-04997-8)
Supplement: Supplementary file 1 — Additional file 1: Table S1. List of formulations with details about active ingredients, their concentration, physical form, and details of the respective solvent per assay. [file 13071_2021_4997_MOESM1_ESM.docx]

**Table S1.** List of formulations with details about active ingredients, their concentration, physical form, and details of the respective solvent per assay.

| **Compound** | **Active ingredients** | **Concentration** | **Form** | **Initial solvent** | **Adult bioassay solvent** | **Larval bioassay solvent** |
| --- | --- | --- | --- | --- | --- | --- |
| Fludora*®* Fusion WP-SB | Deltamethrin | 6.25% | Powder | Water | Ethanol | Water |
|  | Clothianidin | 50% |  |  |  |  |
| K-Othrine*®* WG | Deltamethrin | 25% | Powder | Water | Ethanol | Water |
| Clothianidin WG | Clothianidin | 70% | Powder | Water | Acetone | Water |
| Transfluthrin EC | Transfluthrin | 62.5g/L | Liquid | Water | Methanol | Water |
